# Pulmonary infection in traumatic brain injury patients undergoing tracheostomy: predicators and nursing care

**DOI:** 10.1186/s12890-022-01928-w

**Published:** 2022-04-07

**Authors:** Xuelian Zhang, Hui Zhou, Hongying Shen, Mingli Wang

**Affiliations:** Department of Neurosurgery, The First Affiliated Hospital of Soochow University, No. 188 Shizi Street, Suzhou, Jiangsu China

**Keywords:** Pulmonary infection, Traumatic brain injury, Tracheostomy, Management, Nursing, Care

## Abstract

**Background:**

Pulmonary infection is common yet serious complication in patients with severe traumatic brain injury (STBI). We aimed to evaluate the predicators of pulmonary infection in STBI patients undergoing tracheostomy, to provide evidence for the clinical nursing care of STBI patients.

**Methods:**

This study was a retrospective cohort design. STBI patients undergoing tracheostomy treatment from January 1, 2019 to August 31, 2021 in our hospital were included. The characteristics of pulmonary infection and no pulmonary infection patients were analyzed.

**Results:**

A total 216 STBI patients undergoing tracheostomy were included, the incidence of pulmonary infection was 26.85%. Diabetes (r = 0.782), hypoproteinemia (r = 0.804), duration of coma(r = 0.672), duration of mechanical ventilation(r = 0.724) and length of hospital stay (r = 0.655), length of hospital stay post tracheostomy (r = 0.554), mortality (r = 0.598) were all correlated with pulmonary infection (all *p* < 0.05). *Klebsiella pneumoniae* (33.87%) and *Staphylococcus aureus* (29.03%) were the most commonly seen pathogens in the pulmonary infection of TBI patients. Logistic regression analyses indicated that diabetes (OR 2.232, 95% CI 1.215–3.904), hypoproteinemia with plasma total protein < 60 g/L (OR 1.922, 95% CI 1.083–3.031), duration of coma ≥ 22 h (OR 2.864, 95% CI 1.344–5.012), duration of mechanical ventilation ≥ 5 days (OR 3.602, 95% CI 1.297–5.626), length of hospital stay ≥ 21 days (OR 2.048, 95% CI 1.022–3.859) were the risk factors of pulmonary infection in TBI patients undergoing tracheostomy (all *p* < 0.05).

**Conclusions:**

Further investigations on the early preventions and treatments targeted on those risk factors are needed to reduce the pulmonary infection in clinical practice.

## Background

Severe traumatic brain injury (STBI) is one of the most important causes of death and disability worldwide [1]. Studies [2–4] have found that organ failure and secondary infection after brain trauma are the most important causes of death. The literature reports [5, 6] a fatality rate that can reach 50.17%, and the concurrent pulmonary infection rate can be as high as 60.84%. STBI patients are prone to secondary pulmonary infection, which can significantly increase the disability and mortality of patients [7, 8]. Therefore, early prediction, diagnosis and early intervention of whether STBI patients have secondary pulmonary infection can help to reduce the mortality of patients and improve the prognosis of patients.

During the treatment of STBI, tracheostomy is often used to provide respiratory support in patients with craniocerebral injury [9]. However, tracheostomy is a traumatic operation, which destroys the body barrier of the patient’s respiratory system and it usually takes a long treatment period [8, 10, 11]. Therefore, the STBI patients are more prone to pulmonary infection [12]. Currently, the characteristics of pulmonary infection in STBI patients undergoing tracheostomy remains unclear [13]. Identifying the risk factors of pulmonary infection in STBI patients undergoing tracheostomy is beneficial to the counteractive measures making to reduce pulmonary infection which will impact on the management and treatment of STBI. Therefore, in this present study, we aimed to analyze the characteristics of pulmonary infection in STBI patients undergoing tracheostomy, to identify the potential influencing factors of pulmonary infections, thereby providing reliable evidences into the clinical STBI treatment.

## Methods

### Ethical considerations

In this study, all methods were performed according to the relevant guidelines and regulations. This present study was a retrospective cohort design. And the study had been approved by the ethical committee of First Affiliated Hospital of Soochow University (approval number: 2019-200), and written informed consents had been obtained from all the included patients or related guardians of patient.

### Patients

This study selected STBI patients who underwent tracheostomy in the department of neurosurgery of our hospital from January 1, 2019 to August 31, 2021 as study population. The inclusion criteria were: patients with TBI and a Glasgow Coma Scale (GCS) ≤ 8 points at admission; STBI patients who received tracheostomy treatment during the hospital stay; and patients with duration of mechanical ventilation > 48 h. The exclusion criteria of this study are: patients whose condition deteriorated rapidly and died within 24 h of admission; Patients with pulmonary infection diagnosed by the respiratory symptoms, blood gas analysis and lung X-rays at the admission of patients; patients whose relatives did not agree to participate in this study.

### Diagnostic criteria for pulmonary infection

We focused on the pulmonary infection in STBI patients undergoing tracheostomy, which should be a part of hospital-acquired pneumonia. The diagnosis of pulmonary infection during hospital stay was referred to the relevant diagnostic criteria for pulmonary infection [14–16], specifically as following: ① The patient had respiratory symptoms such as cough, excessive sputum, purulent sputum, rapid breathing, fever; ② body temperature ≥ 38 °C; ③ the white blood cell > 10 × 10^9^; ④ Auscultation of the lungs could hear dry and wet rales; ⑤ X-rays or chest CT of lungs indicated small patchy shadows; ⑥ Pathogenic bacteria cultured in sputum; ⑦ Patients have history of tracheostomy or intubation, aspiration, pulmonary edema, atelectasis, shock, surgical anesthesia, invasive infection of the wound, suppurative thrombophlebitis. Patients can be diagnosed with pulmonary infection if they meet at least 4 items above.

### Bacteriological analysis

We analyzed the bacteria and fungi in the bacteriological detections. We used the Microstation automatic microbial identification system (X900) produced by Henrui Biolog company (Nanjing, China) and related media to configure the bacterial suspension for the identification of pathogenic bacteria, which could automatically generate identification results. Sputum culture device were used to suck the deep secretions of lower respiratory tract through tracheal cannula and immediately sent to our laboratory for bacteria culture and drug sensitivity test. The operation process and the collection of sputum specimens were strictly performed in accordance with the principle of sterility.

### Data collection

We retrospectively collected following data from the medical record: gender, age, body mass index (BMI), alcohol drinking, smoking, hypertension, diabetes, hyperlipidemia, hypoproteinemia (plasma total protein < 60 g/L), types of TBI, GCS at admission, duration of coma, duration of mechanical ventilations, length of hospital stay and mortality. The characteristics of pulmonary infection and no pulmonary infection patients were compared.

### Statistical analysis

We used SPSS 19.0 software to analyze the data. For normally distributed data, we used mean and standard deviation, whereas for non parametric we used median and inter-quartile range (IQR). Comparison between groups was performed by t test. Categorical variables were presented as cases or percentage (%), the group comparisons were performed by chi-square test. Pearson correlation analyses were conducted to assess the correlation of characteristics and pulmonary infection. Logistic regression analyses were conducted to analyze the risk factors related to the occurrence of pulmonary infection. In this study, *P* < 0.05 was considered statistically significant.

## Results

### The characteristics of included patients

A total 216 STBI patients undergoing tracheostomy were included, of whom 58 patients had pulmonary infection, the incidence of pulmonary infection was 26.85%. As presented in Table 1, there were significant differences in the diabetes, hypoproteinemia, duration of coma, duration of mechanical ventilation and length of hospital stay, length of hospital stay post tracheostomy, mortality between pulmonary infection and no pulmonary infection group (all *p* < 0.05). No significant differences in the gender, age, BMI, alcohol drinking, smoking, hypertension, hyperlipidemia, types of TBI, GCS at admission and duration of mechanical ventilation prior to tracheostomy were found (all *p* > 0.05).

**Table 1:** The characteristics of included patients (n = 216)

Variables	Infection group (n = 58)	No-infection group (n = 158)	t/χ^2^	*p*
Male/female	36/22	89/69	1.237	0.065
Age (y)	43.26 ± 6.94	43.92 ± 9.03	7.183	0.121
BMI (kg/m^2^)	23.39 ± 1.09	24.01 ± 1.45	5.246	0.082
Alcohol drinking	38 (65.51%)	91 (57.59%)	1.132	0.067
Smoking	21 (36.21%)	56 (35.44%)	1.088	0.105
Hypertension	33 (56.89%)	88 (55.69%)	1.235	0.071
Diabetes	28 (48.28%)	40 (25.32%)	1.102	0.013
Hyperlipidemia	14 (24.14%)	35 (22.15%)	1.976	0.097
Hypoproteinemia	27 (46.55%)	30 (18.99%)	1.209	0.002
Types of TBI			1.194	0.064
Brain contusion	29 (50.00%)	72 (45.57%)		
Subdural hematoma	17 (29.31%)	45 (28.48%)		
Epidural hematoma	12 (20.69%)	41 (25.95%)		
GCS at admission	5.87 ± 2.15	6.09 ± 2.23	1.556	0.081
Duration of mechanical ventilation prior to tracheostomy (h)	14.91 ± 4.06	14.55 ± 5.13	2.059	0.102
Duration of coma (h)	28.14 ± 7.39	16.11 ± 5.53	4.132	0.001
Duration of mechanical ventilation (days)	6.26 ± 3.43	3.85 ± 2.01	2.206	0.033
Length of hospital stay (days)	24.16 ± 4.14	17.09 ± 3.42	5.097	0.028
Length of hospital stay post tracheostomy (days)	21.05 ± 3.77	14.26 ± 2.86	2.305	0.045
Death	13 (22.41%)	11 (6.97%)	1.018	0.005

### Correlation analysis

As indicated in Table 2, Pearson correlation analyses indicated that diabetes (r = 0.782), hypoproteinemia (r = 0.804), duration of coma (r = 0.672), duration of mechanical ventilation (r = 0.724) and length of hospital stay (r = 0.655), length of hospital stay post tracheostomy (r = 0.554), mortality (r = 0.598) were all correlated with pulmonary infection (all *p* < 0.05).

**Table 2:** Pearson correlation analysis of characteristics and pulmonary infection

Variables	r	*p*
Gender	0.124	0.088
Age (y)	0.211	0.092
BMI (kg/m^2^)	0.161	0.074
Alcohol drinking	0.107	0.115
Smoking	0.244	0.079
Hypertension	0.175	0.103
Diabetes	0.782	0.033
Hyperlipidemia	0.121	0.079
Hypoproteinemia	0.804	0.014
Types of TBI	0.626	0.011
GCS at admission	0.239	0.075
Duration of mechanical ventilation prior to tracheostomy (h)	0.599	0.026
Duration of coma (h)	0.672	0.012
Duration of mechanical ventilation (days)	0.724	0.003
Length of hospital stay (days)	0.655	0.037
Length of hospital stay post tracheostomy (days)	0.554	0.041
Death	0.598	0.029

### Distribution of pathogens

Table 3 indicated the distribution of pathogens of pulmonary infection in STBI patients, *Klebsiella pneumonia* (33.87%) and *Staphylococcus aureus* (29.03%) were most commonly seen pathogens in the pulmonary infection of TBI patients.

**Table 3:** Distribution of pathogens of pulmonary infection in STBI patients (n = 62)

Pathogens	Number	Proportion (%)
Gram-positive bacteria	27	43.55
*Staphylococcus aureus*	18	29.03
*Hemolytic streptococcus*	5	8.06
*Staphylococcus epidermidis*	2	3.23
*Enterococcus faecalis*	2	3.23
Gram-negative bacteria	32	51.61
*Klebsiella pneumoniae*	21	33.87
*Pseudomonas aeruginosa*	5	8.06
*Acinetobacter baumannii*	4	6.45
*Escherichia coli*	2	3.23
Fungus	3	4.84
*Candida albicans*	3	4.84

### Resistant rate and antibiotic sensitivity of *Klebsiella pneumoniae* and *Staphylococcus aureus*

Table 4 indicated the resistant rate and antibiotic sensitivity of *Klebsiella pneumoniae* and *Staphylococcus aureus* to antibiotics. Both *Klebsiella pneumoniae* *Staphylococcus aureus* were highly sensitive to Cefoperazone, Meropenem, and Levofloxacin.

**Table 4:** The resistant rate and antibiotic sensitivity of *Klebsiella pneumoniae* and *Staphylococcus aureus* to antibiotics

Antibiotics	*Klebsiella pneumoniae* to antibiotics (n = 21)			*Staphylococcus aureus* to antibiotics (n = 18)		
	Cases	Resistant rate (%)	Antibiotic sensitivity (%)	Cases	Resistant rate (%)	Antibiotic sensitivity (%)
Levofloxacin	3	7.48	89.55	2	2.55	90.19
Gentamicin	19	73.12	13.67	9	39.52	55.14
Cefepime	4	20.98	76.42	4	10.16	78.34
Piracetam	5	21.04	71.21	3	9.57	87.16
Cefoperazone	1	10.94	93.28	3	2.53	91.82
Ampicillin	12	78.53	20.31	9	54.22	31.91
Meropenem	1	9.10	91.74	2	7.49	90.72
Sulfamethoxazole	11	74.16	22.14	13	67.91	26.34
Cefoxitin	3	8.73	82.95	2	4.84	86.07
Amoxicillin	15	66.97	31.24	12	74.12	11.24
Ceftriaxone	17	63.73	22.01	10	58.34	40.78
Norfloxacin	4	9.04	87.19	2	4.33	84.54

### The risk factors of pulmonary infection in TBI patients undergoing tracheostomy

The variable assignment of multivariate logistic regression in this present study were presented in Table 5. As showed in Table 6, the logistic regression analyses indicated that diabetes (OR 2.232, 95% CI 1.215–3.904), hypoproteinemia (OR 1.922, 95% CI 1.083–3.031), duration of coma ≥ 22 h (OR 2.864, 95% CI 1.344–5.012), duration of mechanical ventilation ≥ 5 days (OR 3.602, 95% CI 1.297–5.626), length of hospital stay ≥ 21 days (OR 2.048, 95% CI 1.022–3.859) were the risk factors of pulmonary infection in TBI patients undergoing tracheostomy (all *p* < 0.05).

**Table 5:** The variable assignment of multivariate logistic regression

Factors	Variables	Assignment
Pulmonary infection	Y	Yes = 1, no = 2
Diabetes	X_1_	Yes = 1, No = 2
Hypoproteinemia	X_2_	Yes = 1, No = 2
Duration of coma (h)	X_3_	≥ 22 = 1, < 22 = 2
Duration of mechanical ventilation (days)	X_4_	≥ 5 = 1, < 5 = 2
Length of hospital stay (days)	X_5_	≥ 21 = 1, < 21 = 2

**Table 6:** The logistic regression analysis on the risk factors of pulmonary infection in STBI patients undergoing tracheostomy

Variables	β	S${\overline{\text{x}}}$	OR	95% CI	*p*
Diabetes	0.321	0.109	2.232	1.215–3.904	0.026
Hypoproteinemia	0.212	0.101	1.922	1.083–3.031	0.041
Duration of coma ≥ 22 h	0.102	0.082	2.864	1.344–5.012	0.013
Duration of mechanical ventilation ≥ 5 days	0.436	0.106	3.602	1.297–5.626	0.011
Length of hospital stay ≥ 21 days	0.123	0.112	2.048	1.022–3.859	0.017

## Discussions

STBI is one of the clinical critical illnesses, which can lead to a higher disability rate and fatality rate. In clinical practice, tracheostomy may reduce the risk of VAP, avoid the complications of prolonged ventilation, and it helps in weaning patients from the ventilator if they are on the ventilator for a prolonged period. However, tracheostomy is one of the high-risk factors for pulmonary infection [17, 18]. However, if patients with TBI are complicated with pulmonary infections, it may further affect their recovery effect and prognosis, and even lead to an increase in their mortality [19]. Therefore, it is very important for such patients to prevent and control pulmonary infections [20, 21]. To achieve effective control and treatment, a comprehensive understanding of associated factors is necessary for the prevention and treatment of pulmonary infection. The results of this study have showed that the incidence of pulmonary infection is 26.85% in STBI patients undergoing tracheostomy. Diabetes, hypoproteinemia, duration of coma ≥ 22 h, duration of mechanical ventilation ≥ 5 days, length of hospital stay ≥ 21 days are the risk factors of pulmonary infection, early preventions and interventions targeted on those risk factors are needed in clinical tracheostomy nursing care.

After tracheostomy, the gas is not humidified and filtered by the upper respiratory tract thus entering the lungs directly, causing possible damage to the ciliary epithelium of the respiratory tract mucosa with difficult removal of secretions and foreign bodies [22, 23]. The patients included in this study received mechanical ventilation after tracheostomy performance, thus receiving active or passive humidification of the airways. The factors that dry inhaled air, blockage of sputum, pathogenic bacteria and particles with pathogenic bacteria make it easy to cause pulmonary infection [24]. At the same time, patients with impaired consciousness who are not ventilated initially post STBI will be vulnerable or prone to aspiration of stomach contents, and digestive juice damages the bronchial mucosa and cilia and may predispose to infection and a picture of pneumonitis [25, 26]. Patients with impaired consciousness are prone to aspiration of stomach contents, and digestive juice damages the bronchial mucosa and cilia and causes infection [27]. In addition, patients with STBI often have stress ulcers, poor gastrointestinal function, malnutrition, which is associated with the occurrence of pulmonary infection [28].

The immune status and body repair ability of diabetic patients are worse than those of no diabetic patients, and the risk of infection is higher [29, 30]. Therefore, intervention on the immune status and blood sugar of diabetic patients should be strengthened [31]. Besides, the longer the coma, the higher risk of pulmonary infection, which is related to the worse immune status [32]. The decline in pulmonary function is also an important reason for the infection [33]. Therefore, interventions in the respiratory tract should be strengthened. People with chronic underlying diseases have relatively poor overall body status, and have poor resistance and repair ability to pathogenic bacteria, so they are more prone to pulmonary infections [34]. Therefore, in addition to the necessary adjustments to the immune status of such patients, close monitoring of blood glucose fluctuations and intensified respiratory management are necessary treatments [35].

In this study, the sputum of patients was cultured and identified, and the types and distribution of pathogens were analyzed. The research results show that gram-negative bacteria are still the first pathogens of lung infections, second by gram-positive bacteria and fungi, suggesting that pathogen monitoring should be strengthened and targeted medications should be given during treatment. Due to long-term exposure to tracheostomy and disease stress, the patient’s immunity is significantly reduced, resulting in a significant increase in the patient’s pulmonary infection rate, and the types of infections are gradually diversified. We have found that *Klebsiella pneumoniae* and S*taphylococcus aureus* were most commonly seen pathogens in the pulmonary infection of TBI patients, both *Klebsiella pneumoniae* and *Staphylococcus aureus* are highly sensitive to Cefoperazone, Meropenem, and Levofloxacin. Therefore, clinical antibiotics should be given according to the drug resistance and sensitivity assessment, to improve the effects and safety of treatment [36, 37].

Pulmonary infection seriously affects the prognosis of patients with STBI, and infection should be prevented and controlled in a timely and effective manner [38]. The clinical treatment and nursing operations should strictly abide by the aseptic operating procedures, including tracheostomy, dressing change, and tracheal tube care throughout the entire process. Meanwhile, sputum bacteria culture + drug susceptibility test should be carried out in time, antibiotics should be adjusted in time according to the test results, and the collection of sputum specimens should avoid contamination that affects the test results and delays treatment [39]. Suction sputum in time, try to avoid infection around the tracheal incision and damage to the mucous membrane of the respiratory tract to reduce bacterial migration [40, 41]. Besides, adopt a reasonable posture, turn over and pat the back on time to avoid sputum accumulation is helpful to prevent pneumonia [42–44]. Furthermore, early nutritional supports are needed, and if necessary, infusion of plasma or albumin to improve the body's resistance to prevent and reduce the chance of nosocomial infection [45].

Several limitations in this present study must be considered. Firstly, our study is a retrospective study design, the collected data are very limited, there can be other factors influencing pulmonary infection that we cannot include for analysis. Secondly, even rough the sample size is large enough, it can be underpowered for some factors to detect the group differences, there by potential biases can be existed. Thirdly, the most included STBI were adult patients with age ≥ 18 years, only 4 children with STBI were included in our study, the further stratified analysis of pulmonary infection in the adult and children populations are needed in the future with larger sample size and rigorous design.

## Conclusions

In conclusion, we have found that the pulmonary infection is a very common and serious complication in patients undergoing tracheostomy. Diabetes, hypoproteinemia, duration of coma ≥ 22 h, duration of mechanical ventilation ≥ 5 days, length of hospital stay ≥ 21 days are the risk factors of pulmonary infection in those patients. And the pulmonary infections are mainly caused by gram-negative bacteria. Future studies on the effects and safety of timely and effective measures in response to these risk factors to reduce the pulmonary infections and improve the prognosis of STBI patients are needed.

## Data Availability

All data generated or analyzed during this study are included in this published article.

## Abbreviations

STBI: Severe traumatic brain injury
GCS: Glasgow Coma Scale
BMI: Body mass index
